# Supplementary material for: Content-rich biological network constructed by mining PubMed abstracts
Source: BMC Bioinformatics. 2004 Oct 8;5:147. doi: 10.1186/1471-2105-5-147 (PMC528731; doi:10.1186/1471-2105-5-147)
Supplement: Additional File 2 — The original results of the above study (non-essential files are deleted to keep the file size under the limit set by BMC bioinformatics). [file 1471-2105-5-147-S2.bz2 › chilibotAdditionalFile2/dip40/540ID9211894E10893/html/AR_FOS.html]

 


 **AR** and **FOS** 
  
Found 62 abstracts in PubMed, retrieved 40.  
 

 What does Google say? 
 PDF only 
| .edu only 

---

**Interactive relationship** (e.g. stimulation, inhibition, etc)

**Neutral relationship**- Neurons in these brain areas are rich in androgen receptors  **AR**  and express  **FOS**  immunoreactivity in response to mating.  Ref: 12689608 Psychoneuroendocrinology, 2003
- Androgen receptor  [ **AR** ]  immunoreactivity and mating induced  **Fos**  expression in forebrain and midbrain structures in the male rat.  Ref: 8923531 Neuroscience, 1996
- However, not all androgen receptor  [ **AR** ]  neurons were  **Fos**  expressive, suggesting that only some androgen sensitive neurons were activated during mating.  Ref: 8923531 Neuroscience, 1996
- The  **AR**  min group showed the greatest difference in  **Fos**  lir compared with the MR groups.  Ref: 12492299 Behav Neurosci, 2002
- The distributions of neurons containing androgen receptors  **AR**  and estrogen receptors ER overlap, and many  **AR**  immunoreactive  **AR**  ir neurons express  **Fos**  immunoreactivity  **Fos**  ir induced by mating.  Ref: 9485165 Neuroendocrinology, 1998
- In these regions mating induced  **Fos**  immunofluorescence was exclusively localized in androgen receptor  [ **AR** ]  immunofluorescent neurons.  Ref: 8923531 Neuroscience, 1996
- **Fos**  was also localized in subsets of  **AR**  ir neurons within the L5, L6, and S1 segments of the spinal cord.  Ref: 9647938 Horm Behav, 1998
- EMSA supershift experiments and GST  **AR**  pull down experiments followed by Western blotting identified a  **Fos**  related protein of approximately 40 kDa as part of this complex.  Ref: 9241247 Nucleic Acids Res, 1997
- **Fos**  was highly localized in subsets of  **AR**  ir neurons within the medial preoptic nucleus, bed nucleus of the stria terminalis, dorsomedial nucleus of the amygdala, and central tegmental field.  Ref: 9647938 Horm Behav, 1998
- Two studies were designed to document neuronal colocalization of androgen receptor  [ **AR** ]  immunoreactivity and mating induced  **Fos**  immunoreactivity  **AR**  ir,  **Fos**  ir in brain of male rats and to examine the extent to which limbic and midbrain neurons that project to the preoptic area are androgen sensitive and activated by mating.  Ref: 9507053 Brain Res, 1998
- These results suggest that elevation of c  **fos**  expression is associated with a decrease in  **AR**  and mRNA and provide correlative data supporting negative repression by c  **fos**  on androgen receptors levels.  Ref: 9027330 Mol Cell Endocrinol, 1996
- Spinal cord concentrations of  **AR**  ir and  **Fos**  ir neurons were greatest in Lamina X, and the vast majority of  **Fos**  ir neurons in the dorsal part of Lamina X were also  **AR**  ir.  Ref: 9647938 Horm Behav, 1998
- The distribution of androgen receptor  [ **AR** ]  immunoreactive neurons, mapped with the PG21 anti androgen receptor  [ **AR** ]  antibody, was compared in male rat brains with the distribution of  **Fos**  immunoreactive neurons induced by mating.  Ref: 8923531 Neuroscience, 1996
- These results indicate that a  **Fos**  containing protein complex distinct from AP 1 binds upstream of the AREs in the PSA and KLK2 promoters, interacts with the  **AR**  and may participate in regulation of these two androgen responsive genes.  Ref: 9241247 Nucleic Acids Res, 1997
- Compared with MR rats, pup exposed  **AR**  rats engaged in less pup licking, and all  **AR**  rats showed significant reductions in c  **fos**  immunoreactivity in the medial preoptic area and the parietal and piriform cortices.  Ref: 12492299 Behav Neurosci, 2002
- Pre treatment with the selective beta 3  **AR**  antagonist SR59230A resulted in a significant decrease in the number of  **Fos**  positive cells in all those areas compared with rats treated with CL316243 alone.  Ref: 10963888 Neurosci Lett, 2000
- In addition to its ability to activate transcription from androgen response elements,  **AR**  can inhibit activator protein 1 AP 1 activity, composed of Jun and  **Fos**  oncoproteins, in a ligand dependent manner.  Ref: 9822653 J Biol Chem, 1998
- c  **Fos**  dimerization with c Jun represses c Jun enhancement of androgen receptor  [ **AR** ]  transactivation.  Ref: 9867253 Endocrine, 1998

**Non-interactive relationship** (e.g. studied together, co-existance, homology, etc.)

- In dMEA and CTF, nuclear colocalization of  **AR**  ir and mating induced  **Fos**  ir was present in a proportion of FG containing neurons.  Ref: 9507053 Brain Res, 1998
- After testing, brains and spinal cords were examined for  **AR**  and  **Fos**  immunoreactivity ir.  Ref: 9647938 Horm Behav, 1998
- There is now evidence for a critical cell signaling pathway, regulated by c  **fos**  expression, necessary for castration induced apoptosis, as well as evidence that this signaling initiates an abrupt and transient alteration in the synthesis of fas antigen, p53, bax and bcl 2 proteins in the androgen receptor  [ **AR** ]  expressing prostate epithelial cells, the cellular compartment that appears to be the most affected by castration.  Ref: 10322394 Trends Endocrinol Metab< MedlineTA>Trends Endocrinol Metab, 1998
- and alpha1  **AR**  mediated c  **fos**  expression by Northern blot analysis.  Ref: 9285642 Circ Res, 1997
- Androgen receptor  [ **AR** ]  and mating induced  **fos**  immunoreactivity are co localized in limbic and midbrain neurons that project to the male rat medial preoptic area.  Ref: 9507053 Brain Res, 1998
- Because mating induced  **Fos**  ir in the male rat occurs mainly in  **AR**  ir neurons, and because both steroids are important for mating, we hypothesized that i  **AR**  ir and ER ir are colocalized.  Ref: 9485165 Neuroendocrinology, 1998
- 1,  **Fos**  ir and  **AR**  ir were colocalized in neurons of the medial preoptic nucleus MPN, the dorsal medial amygdala dMEA, the central tegmental field CTF, the bed nucleus of the stria terminalis, the anterior hypothalamus, the lateral hypothalamus, and the ventral premamillary nucleus.  Ref: 9507053 Brain Res, 1998
- This study was designed to determine the extent to which  **Fos**  immunoreactivity induced either by mating or noncontact sociosexual interaction and androgen receptor  [ **AR** ]   **AR**  immunoreactivity are colocalized in brain and spinal cord of male rats.  Ref: 9647938 Horm Behav, 1998
- The alpha 2  **AR**  agonists, clonidine and UK14304 also had no effect on basal IEG levels, while blockade of alpha 2 ARs by methoxyidazoxan significantly increased NGFI A and c  **fos**  expression, but decreased c jun mRNA levels.  Ref: 11072104 Brain Res Mol Brain Res, 2000
- Ang II selectively downregulates alpha1a  **AR**  subtype mRNA and its corresponding receptor as well as alpha1a  **AR**  mediated expression of the immediate early gene c  **fos**  in cardiac myocytes.  Ref: 9285642 Circ Res, 1997
- Alpha1  **AR**  stimulated c  **fos**  induction, which could be blocked by 5 methylurapidil but not by chloroethylclonidine, was attenuated by Ang II preincubation.  Ref: 9285642 Circ Res, 1997
- Here, we report that alpha 1 B ARs inhibit the activities of alpha 1A ARs in neonatal rat myocardium so that the inactivation of alpha 1 B ARs by chloroethylclonidine CEC potentiated the effects of nonselective alpha 1  **AR**  agonist phenylephrine PE on myocardial protein synthesis and early gene c  **fos**  and c jun expression.  Ref: 9655894 J Pharmacol Exp Ther, 1998
- In a second experiment, we used immunofluorescent techniques to document the intraneuronal co localization of  **Fos**  with androgen receptor  [ **AR** ]  immunoreactivity in the medial preoptic area, medial amygdala, and central tegmental field.  Ref: 8923531 Neuroscience, 1996
- Steady state  **AR**  mRNA levels increased significantly by the age of 85 days while c  **fos**  mRNA levels remained at low baseline levels at all ages.  Ref: 9027330 Mol Cell Endocrinol, 1996
- We have conducted experiments investigating the effects of i.c.v. administration of the selective beta 3  **AR**  agonist CL316243 on  **Fos**  expression to determine whether beta 3  **AR**  stimulation affects neurones within specific brain nuclei.  Ref: 10963888 Neurosci Lett, 2000
- We have previously reported an indirect mechanism by which c Jun is able to regulate transcription by stimulating androgen receptor  [ **AR** ]  transactivation in the absence of c  **Fos**  or any apparent DNA binding.  Ref: 9591784 Oncogene, 1998
- PG21 anti  **AR**  and anti c  **fos**  primary antibodies were visualized by fluorescence microscopy using cyanine conjugated and fluorescein conjugated secondary antibodies.  Ref: 9507053 Brain Res, 1998
- Although the AP 1 c Jun c  **Fos**  system and NcoR have been reported to interact with and repress the activity of some steroid receptors, c Jun, c  **Fos** , c Jun c  **Fos** , nor NcoR function was consistently affected by the absence or presence of residues 628 646, implying that the  **AR**  hinge region exerts its silencing effects in a manner independent of these corepressors.  Ref: 11102454 J Biol Chem, 2001
- Brains from male rats, killed 1 h after ejaculating with receptive females, were examined for  **Fos**  ir and  **AR**  ir and compared with those from control rats not given access to females.  Ref: 9507053 Brain Res, 1998
- Since activator protein 1 AP 1, a heterodimer of c jun and c  **fos**  proteins, is known to inhibit binding of  **AR**  to ARE by protein protein interaction with  **AR** , the findings in the present study suggest a possible involvement of AP 1 in the antiandrogenic effects of PAHs acting as AhR agonists.  Ref: 12799773 Arch Toxicol, 2003
- **AR**  ir and  **Fos**  ir were visualized with fluorescence microscopy using cyanine conjugated and fluorescein conjugated secondary antibodies.  Ref: 10333272 J Comp Neurol, 1999
- PG21 anti  **AR** , OA 11 824 anti c  **fos** , H222 or 1D5 anti ER primary antibodies were visualized, respectively, with cyanine conjugated, fluorescein or cyanine conjugated, and fluorescein conjugated secondary antibodies in male rats which were killed 1 h after ejaculating with a receptive female.  Ref: 9485165 Neuroendocrinology, 1998
- After experiencing social stress the brain area within the medial preoptic area was removed for analysis of c  **fos**  and androgen receptor  [ **AR** ]  mRNA expression.  Ref: 12131378 J Urol, 2002
- Brains were subsequently examined for FG transport,  **Fos**  ir and  **AR**  ir.  Ref: 9507053 Brain Res, 1998
- Brains and spinal cords were examined for FG transport,  **AR**  immunoreactivity  **AR**  ir, and  **Fos**  immunoreactivity  **Fos**  ir.  Ref: 10333272 J Comp Neurol, 1999
- ... inhibition of c  **Fos** , c Jun, HB EGF,  **AR** , and VEGF mRNA by this compound was incomplete 41 65%.  Ref: 12016209 J Biol Chem, 2002
- We report that, identically to 17beta estradiol or dihydrotestosterone, but differently from raloxifene, estren alters the activity of Elk 1, CCAAT enhancer binding protein beta C EBPbeta, and cyclic adenosine monophosphate response element binding protein CREB, or c Jun c  **Fos**  by an extranuclear action of the ER or  **AR** , resulting in activation of the Src Shc ERK pathway or downregulation of JNK, respectively.  Ref: 12782668 J Clin Invest, 2003
- PG21 anti  **AR**  and anti c  **fos**  primary antibodies were visualized by fluorescence microscopy using cyanine conjugated and fluorescein conjugated secondary antibodies.  Ref: 9647938 Horm Behav, 1998
- Effect of prenatal androgen receptor  [ **AR** ]  antagonist or aromatase inhibitor on sexual behavior, partner preference and neuronal  **Fos**  responses to estrous female odors in the rat accessory olfactory system.Many socially relevant odors are detected in rodent species by the vomeronasal organ and subsequently processed by the accessory olfactory system.  Ref: 11897260 Physiol Behav, 2002
- All brain regions in which androgen receptor  [ **AR** ]  immunoreactive neurons were counted also expressed  **Fos**  immunoreactivity after mating, and there was considerable overlap between the distributions of androgen receptor  [ **AR** ]  and  **Fos**  immunoreactive neurons.  Ref: 8923531 Neuroscience, 1996
- Autoregulation of androgen receptor  [ **AR** ]  in rat ventral prostate involvement of c  **fos**  as a negative regulator.  Ref: 9027330 Mol Cell Endocrinol, 1996
- Moreover, the brain dopamine and serotonin balance, but not norepinephrine turnover, was dependent on alpha2C  **AR**  expression, and the expression of c  **fos**  and junB mRNA was increased in alpha2C KO mice.  Ref: 10523817 Mol Psychiatry, 1999
- We examined, in adjacent sections from the medial preoptic area MPN through the central tegmental field CTF, the expression of ER ir in i  **AR**  ir containing neurons, and ii  **Fos**  ir expressive neurons.  Ref: 9485165 Neuroendocrinology, 1998
- Furthermore, androgens appear to maintain androgen receptor  [ **AR** ]  levels and androgen sensitivity by continuous suppression of the repressor, c  **fos** .  Ref: 9027330 Mol Cell Endocrinol, 1996
- This study examined if mating induced  **Fos**  expression a measure of neuronal activation and androgen receptors  **AR**  are colocalized in brain and spinal cord neurons which project to the CTF.  Ref: 10333272 J Comp Neurol, 1999
